# Draft Genome Sequence of *Coralloluteibacterium stylophorae* LMG 29479^T^

**DOI:** 10.1128/MRA.00421-21

**Published:** 2021-07-08

**Authors:** Andrey V. Karlyshev, Ekaterina B. Kudryashova, Elena V. Ariskina, Ava P. Conroy, Elena Y. Abidueva

**Affiliations:** a School of Life Sciences Pharmacy and Chemistry, Faculty of Science, Engineering and Computing, Kingston University London, Kingston upon Thames, United Kingdom; b All-Russian Collection of Microorganisms (VKM), G. K. Skryabin Institute of Biochemistry and Physiology of Microorganisms, Pushchino Scientific Center for Biological Research of the Russian Academy of Sciences, Pushchino, Russia; c RWJBarnabas Health, West Orange, New Jersey, USA; d Institute of General and Experimental Biology, Siberian Branch Russian Academy of Sciences, Ulan-Ude, Russia; Indiana University, Bloomington

## Abstract

Here, we report a draft genome sequence of the strain Coralloluteibacterium stylophorae LMG 29479^T^, acquired from the Belgian Coordinated Collections of Microorganisms. The genus *Coralloluteibacterium* currently includes only one species with a validly published name. These genome sequencing data are important for the phylogeny of the *Lysobacteraceae* family.

## ANNOUNCEMENT

Here, we report a draft genome sequence of Coralloluteibacterium stylophorae LMG 29479^T^ (Sty a-1^T^), isolated from a reef-building coral, *Stylophora* sp., off the coast of Southern Taiwan (1). At the time of writing, the genus *Coralloluteibacterium,* belonging to the family *Lysobacteraceae* (2) of *Gammaproteobacteria*, was represented by *C. stylophorae* as the only species with a validly published name, besides *C. thermopilus* (3), for which the name has not yet been validated (List of Prokaryotic names with Standing in Nomenclature [LPSN], https://lpsn.dsmz.de/).

The culture was grown on LB agar for 24 hours at 37°C; 5 OD_600_ (optical density at 600 nm) units were resuspended in 0.5 ml of DNA/RNA Shield (Zymo Research, Cambridge Bioscience, UK) and 40 μl was used for DNA extraction. After addition of 120 μl of Tris-EDTA (TE) buffer with lysozyme (0.1 mg/ml) and RNase A (0.1 mg/ml), the mixture was incubated for 25 min at 37°C; proteinase K and SDS were added to 0.1 mg/ml and 0.5%, respectively, followed by incubation for 5 min at 65°C. Genomic DNA was purified using an equal volume of SPRI beads (Beckman, USA) and resuspended in elution buffer (EB) solution (Qiagen, Germany). The sequencing DNA library was prepared using the Nextera XT library prep kit (Illumina, San Diego, CA) following the manufacturer’s protocol. Sequencing was performed using the Illumina NovaSeq 6000 system. Reads were adapter trimmed using Trimmomatic 0.30 with a sliding window quality cutoff of Q15 (4). The sequencing produced 2 × 576,649 paired reads (up to 250 bp), which were assembled using SPAdes 3.7 software to produce 29 >1-kb contigs, with a maximum size of 559,068 bp and an *N*_50_ value of 342,650 bp. The assembly size was 4,002,885 bp with 65.12× coverage and GC content of 71.9%.

**FIG 1:**
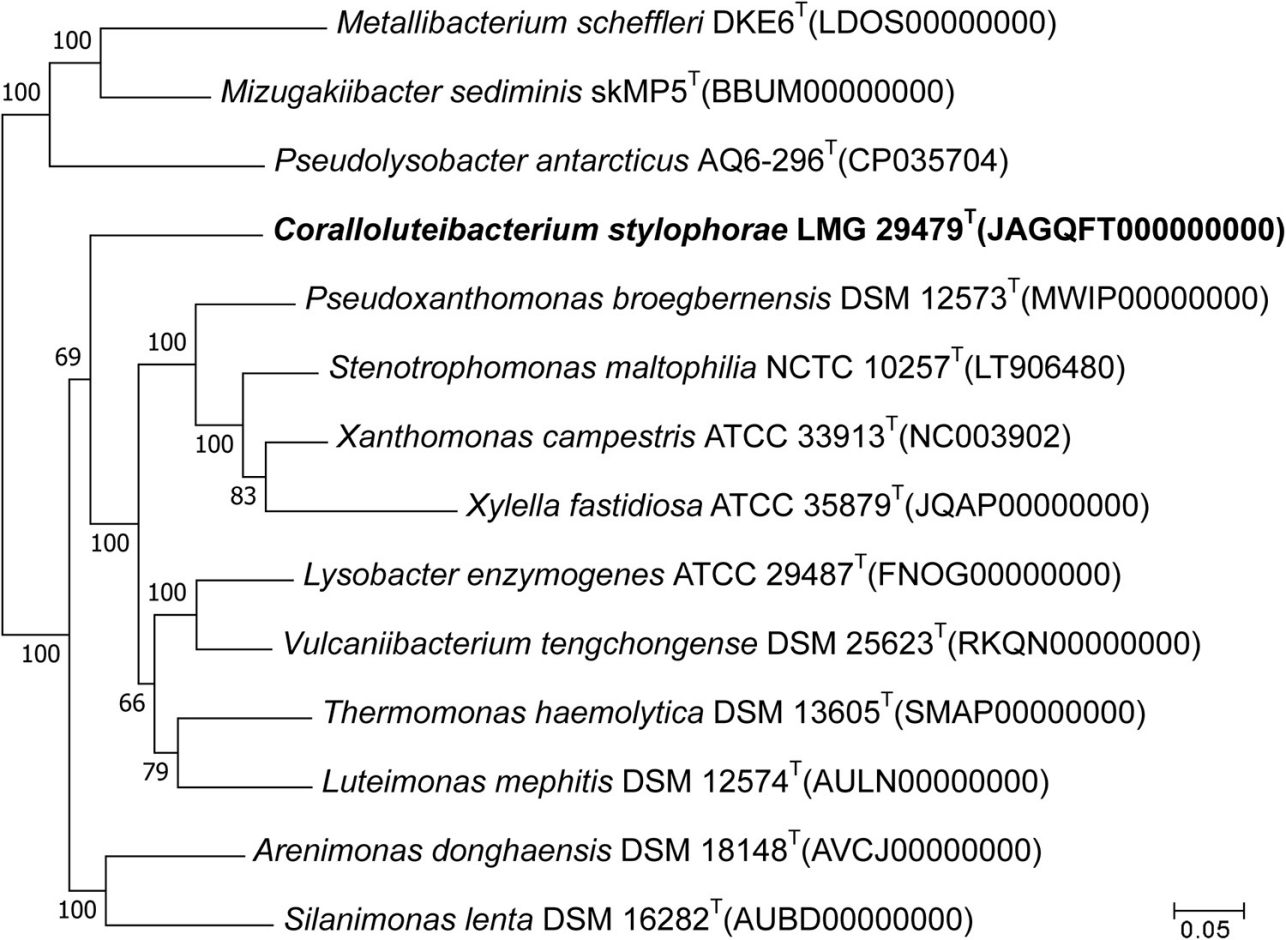
Maximum likelihood tree, showing the phylogenomic position of strain Coralloluteibacterium stylophorae LMG 29479^T^ (JAGQFT000000000) in the family *Lysobacteraceae*, constructed using concatenated alignments of 76 conserved protein sequences using the M1CR0B1AL1Z3R server (https://microbializer.tau.ac.il) (7). Numbers at branch nodes refer to bootstrap values of >50% (100 replicates). Pseudomonas aeruginosa DSM 50071^T^ (CP012001) was used as an outgroup. Bar, 0.05 substitution per nucleotide sequence position.

The results of comparative core genome analysis are provided in Fig. 1. The assembly was submitted to GenBank and annotated using NCBI Prokaryotic Genome Annotation Pipeline (5) v.5.1, using the best-placed reference protein set and GeneMarkS-2+, which identified 3,627 genes, including 3,534 protein-coding genes, 36 pseudogenes, 49 tRNAs, and 1 set of rRNA genes (5S, 16S, and 23S). The analysis revealed the presence of a number of genes putatively involved in osmotic, oxidative, and starvation stress responses. In addition, it has a gene encoding a putative integron integrase. There are a number of genes likely to be involved in sulfur assimilation and in motility/chemotaxis. The organism is capable of producing only one secretion system (type IV). In addition, the strain appears to have the capability of producing type IV pili, which are known to be involved in twitching motility in other bacteria (6). Among the other remarkable features found were the presence of six putative beta-lactamase-encoding genes and 15 genes encoding transposases. Also found were genes potentially involved in the biosynthesis of the resistance-nodulation-division (RND) efflux pump. The results presented in this study will assist in better understanding the adaptation of these bacteria to specific environmental conditions and, as the first report of the genome sequence of bacteria belonging to the genus *Coralloluteibacterium*, will also be of interest to scholars studying microbial taxonomy.

### Data availability.

This whole-genome shotgun project has been deposited at DDBJ/ENA/GenBank under the accession number JAGQFT000000000, BioProject number PRJNA722145, and BioSample number SAMN18746351. The version described in this paper is the second version, JAGQFT020000000. Raw sequences were deposited under SRA accession number SRR14471161.
